# P-2094. Climate Change, Antimicrobial Resistance, and Agricultural Practices: Perspectives from India's Public Health Frontlines

**DOI:** 10.1093/ofid/ofaf695.2258

**Published:** 2026-01-11

**Authors:** Sunitha Chandrasekhar Srinivas, Siri C Peddineni, Vaishnavi RV, Natalie Axelrod, Sanjana Ravi, Venkat Chekuri, Apeksha Kakkar, Maya Muckatira, Gautam Kalyatanda

**Affiliations:** Karuna Trust, Bangalore, Karnataka, India; University of Florida College of Medicine, Jacksonville, FL; Karuna Trust, Bangalore, Karnataka, India; University of Florida College of Medicine, Jacksonville, FL; University of Florida College of Medicine, Jacksonville, FL; Karuna Trust, Bangalore, Karnataka, India; University of Florida Department of Infectious Disease and Global Medicine, Gainesville, Florida; University of Texas Austin, Austin, Texas; University of Florida, Gainesville, Florida

## Abstract

**Background:**

Last-mile healthcare providers (HCPs) are crucial in delivering essential services to rural populations, connecting remote communities to healthcare systems. This study examines HCP perspectives on the impact of climate change on vulnerable populations and identifies opportunities for developing strategies to address these challenges.

**Methods:**

A five-question survey was distributed among 71 Last-Mile Health Care Providers across six Primary Health Care Centers (PHCs) in Karnataka, India. Three of these PHCs were located in rural areas, while the other three were situated in urban areas. The survey addressed topics related to climate change, infection control, and the use of antimicrobials, pesticides, and antifungals in farming.

**Results:**

Among the respondents, 87% were female, averaging 39 years of experience. Of these, 56% were Accredited Social Health Activists (ASHAs), who work in India's public health system. A notable 97% recognized that climate change contributes to antimicrobial resistance (AMR) and is linked to antibiotic use in agriculture and animal husbandry. All respondents employed proper hospital waste disposal methods and believed climate change affects their health, especially when walking long distances in higher temperatures. Most advocated for community and school education programs and suggested creating infographics to raise awareness in the community. They were also aware of antibiotic use in livestock and felt farmers should be educated on reducing pesticides, fertilizers, and antibiotic use for livestock.

**Conclusion:**

The results indicated that healthcare professionals (HCPs) in both rural and urban communities recognize the harmful effects of climate change on their health and the well-being of their communities. Additionally, the findings highlighted the negative impact on the wellness of some last-mile healthcare workers, particularly those from lower socioeconomic backgrounds. The HCPs recommended that future health education initiatives should focus on creating infographics to educate communities about planetary health, sustainable farming practices, and proper waste disposal methods.

**Disclosures:**

All Authors: No reported disclosures

